# The MYRACLE protocol study: a multicentric observational prospective cohort study of patients with multiple myeloma

**DOI:** 10.1186/s12885-019-6080-8

**Published:** 2019-08-29

**Authors:** Lina Benaniba, Benoit Tessoulin, Sabrina Trudel, Catherine Pellat-Deceunynck, Martine Amiot, Stéphane Minvielle, Pierre Antoine Gourraud, Sophie de Visme, Hervé Maisonneuve, Anne Lok, Steven Le Gouill, Philippe Moreau, Cyrille Touzeau

**Affiliations:** 1 Service d’hématologie clinique, Centre Hospitalier Universitaire, Place Alexis Ricordeau, 44093 Nantes, France; 2 CRCINA, INSERM, CNRS, Université d’Angers, Université de Nantes, Nantes, France; 3 Site de Recherche Intégrée sur le Cancer (SIRIC) « ILIAD », INCA-DGOS-Inserm_12558, Nantes, France; 4 Centre d’investigation clinique (CIC) 1413, Inserm, pole hospitalo-universitaire 11 : Santé publique, Clinique des données, Centre Hospitalier Universitaire, Nantes, France; 5 Service d’hématologie clinique, Centre Hospitalier Departemental, La Roche sur Yon, France

**Keywords:** Multiple myeloma, Cohort, Quality of life, Pharmacoeconomy, Epidemiology, Drug resistance, Apoptosis, P53

## Abstract

**Background:**

Despite recent advances in the treatment of multiple myeloma, the disease constantly relapses and is still considered as incurable. The current knowledge about the biological mechanisms underlying resistance to the different class of drugs in multiple myeloma remains poor. The primary objective of the MYRACLE (Myeloma Resistance And Clonal Evolution) cohort, a multicenter prospective cohort of patients with multiple myeloma, is to address this limitation. We here describe the study background, design and methods used for this cohort.

**Methods/design:**

All patients (> 18 year old) diagnosed with de novo or relapsed multiple myeloma and treated in two hematology department from west of France are included in the MYRACLE cohort. Patients provide a signed informed to be included in the study. All subjects are followed until refusal to participate in the study or death. The MYRACLE cohort prospectively collects data on socio-economic status, medical status, imaging, prognosis factors, MM therapies and associated events (resistance, safety issues). Patients also complete standardized quality of life questionnaires. In addition, bone marrow samples will be collected at time of diagnosis and relapses to perform biomarkers analysis and functional assays exploring mechanisms underlying drug resistance.

**Discussion:**

The “real-life” MYRACLE cohort offers the opportunity to prospectively collect epidemiological, medical, QoL and biological data from MM patients during the course of the disease (at time of diagnosis and subsequent relapses). At mid-tem, this integrative cohort will be unique at producing a large variety of data that can be used to conceive the most effective personalized therapy for MM patients. Additionally, the MYRACLE cohort will allow integrating the medical care of MM patients in a health and pharmacoeconomic perspective.

## Background

Multiple myeloma (MM) accounts for 1% of all cancers and nearly 10% of hematological malignancies. The incidence in Europe is 6/100000/year with a median age at diagnosis of 72 years [1]. The disease is characterized by the presence of clonal plasma cells in the bone marrow, leading to anemia, hypercalcemia, renal failure and bone lesions. Major prognosis factors influencing patient outcome are serum levels of albumin, beta2microglobulin, LDH and cytogenetic abnormalities (i.e. 17p deletion and t(4;14) translocation) [2]. The median overall survival of patients with multiple myeloma (MM) has been dramatically improved in the past decades [3]. This progress is the consequence of the wide use of proteasome inhibitors (bortezomib, carfilzomib, ixazomib) and immunomodulatory drugs (thalidomide, lenalidomide and pomalidomide) [4]. More recently, monoclonal antibodies (daratumumab, elotuzumab) and CAR (chimeric antigen receptors) -T cells also led to improve the survival of myeloma patients and immunotherapy therefore became a new cornerstone for myeloma therapy [5, 6]. However, the disease constantly relapses and is still considered as incurable. The prognosis of myeloma patients whose disease is refractory to proteasome inhibitors and immunomodulatory agents still remains very poor [7]. The disease course therefore consists on the alternation of period of response to therapy, disease progression during the subsequent therapies given to the patient. In MM, drug resistance can be schematically divided in two categories: primary resistance (absence of response to the drug) and acquired resistance (initial sensitivity to the drug but occurrence of resistance due to the emergence of resistant subclones) [8]. The Darwinian selective pressure and its underlying biological mechanisms still need to be clarified in MM. Biobanks of tumor samples collected in a standardized manner before starting therapy and at time of relapse are required to achieve this goal. Furthermore, MM therapies have an increasing impact on economy of health. The median annual cost for MM therapies increased from nearly 30,000 to 1,000,000 dollars between 2004 and 2009 in the United States of America [9]. It is of strategic importance to determine predictors of sensitivity to drugs in order to spare patients from unnecessary treatment but also to optimize healthcare resources. Data obtained from a real life prospective MM cohort would help to evaluate this pharmacoeconomic factors. Finally, quality of life (QoL) is an important indicator for cancer patient, both in clinical management and cost effective evaluation of therapies [10]. Outside clinical trials, there are few data describing the influence of different therapies on the QoL of myeloma patients.

To address all these limitations, we have developed a multicenter prospective cohort of patients with MM, the MYRACLE cohort. The present report describes the methodology used to establish this cohort. This study relies on a care and research network including the department of haematology of Nantes, the department of haematology of La Roche sur Yon and the CRCINA (Centre de Recherche en Cancérologie et Immunologie Nantes Angers) center.

## Study objectives

The goals of the MYRACLE cohort are:

- To provide epidemiology data on MM (incidence, temporal trends, geographical and social disparities, risk factors).
- To provide real life data on therapeutic landscape of MM patients (nature of treatments, duration of response to each sequential line of treatment).
- To understand the biological mechanisms associated with drug resistance and clonal evolution in MM.
- To identify new therapeutic targets for the MM treatment.
- To determine the impact of MM therapies on patient quality of life.
- To provide prognostic scores predicting response to MM therapies.
- To conduct pharmaco-economic evaluations of MM therapies.
- To provide a large bone marrow and blood samples biocollection obtained from MM patients at subsequent disease period (diagnosis, reponse to treatment, disease progression).

## Design

### Study design

The cohort MYRACLE is a prospective and observational study including all patients with de novo or relapsed MM diagnosed in two specialized centers (Department of hematology, University hospital, Nantes; department of hematology, La Roche Sur Yon). Patients are followed over at least a period of 10 years. For patients included a time of disease relapse, previous clinical data are recorded retrospectively from medical files. The cohort is registered by the French data protection authority (Commission nationale de l’informatique et des libertés, CNIL) The study is approved by an ethic committee (Comité de Protection des Personnes - Tours - Région Centre - Ouest 1).

### Study population

Adult men and women (> 18 years old) diagnosed with de novo or relapsed MM according to international criteria are eligible for the study [11]. Exclusion criteria include the absence of health insurance and mental condition preventing informed consent. The physician recruits the patient. Once they have been informed, all patients who have read the information letter and give informed consent are included. The data will not be unnamed for the physicians to provide reliable data. However, an identification code will be assigned to every patient for the study of collected samples and the bio-collection.

### Study visits

**Fig. 1:**
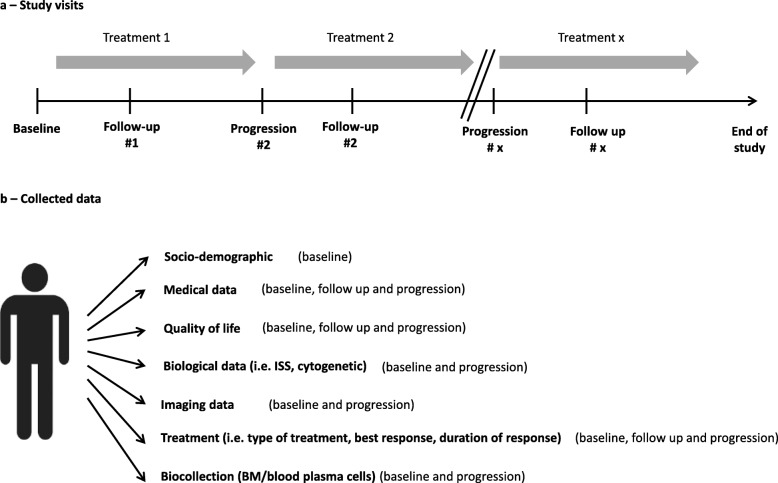
Study visits (**a**) and summary of collected data (**b**) throughout the MYRACLE cohort study. Legend: BM, bone marrow ISS, international scoring system

In the cohort MYRACLE, study visits include: baseline visit, follow up visit (performed 2 months after each treatment initiation) and relapse/progression visit (performed at time of disease progression, under each line of therapy). The flow chart of planned visits is summarized in the Fig. 1. Data collected during each visit are detailed below and in the Table 1.

**Table 1:** Collected data throughout the MYRACLE cohort study

Collected data			Baseline	Follow up	Disease progression
Sociodemographic	Age		x		
	Sex		x		
	Profession		x		
	Place of residence		x		
	Smoking and alcohol habits		x		
	Hematological Family history		x		
Clinical data	Medical family history		x	x	x
	Date of diagnosis		x	x	x
	Anemia		x	x	x
	Renal failure		x	x	x
	Hypercalcemia		x	x	x
	Bone lesion		x	x	x
	Extramedullary disease		x	x	x
Quality of life	SF-36 score		x	x	x
	EQ-5D		x	x	x
Biological data	blood	Blood count	x	x	x
		Creatinine level	x	x	x
		Calcemia	x	x	x
		LDH level	x	x	x
		Albuminemia	x	x	x
		Monoclonal protein amount	x	x	x
	urine	Monoclonal protein amount	x	x	x
		Albuminuria	x	x	x
	Bone marrow	Flow cytometry	x		x
		Cytogenetic {t(11;14), t(4;14), del17p, 1p deletion, 1q gain}.	x		x
		ISS R-ISS	x		x
Imaging	Standard radiography		x		x
	CT scan		x		x
	MRI scan		x		x
	PET scan		x		x
Therapy	Previous lines		x		x
	Best response to therapy		x	x	x
	Duration of response		x	x	x
Biocollection	BM tumor cells		x		x
	Blood tumor cells		x		x

Legend: *SF-36* study short form, *EQ-5D* EuroQOL five dimensions survey, *LDH* lactate dehydrogenase, *ISS* international scoring system, *R-ISS* revised-international scoring system, *CT* computed tomography, *MRI* Magnetic Resonance Imaging, *PET* positon emission tomography, *BM* bone marrow

### Data collection

The MYRACLE study is based on a continuous and comprehensive record of prespecified data obtained from all included patients. Specific data are recorded at baseline and at each follow up visit. The flow chart of planned visits is summarized in the Fig. 1. Data collected during each visit are detailed below and in the Table 1.

#### Sociodemographic and epidemiological data

Sociodemographic and epidemiological characteristics are collected by mean of standardized questionnaires and include age, sex, profession, place of residence, smoking / alcohol habits and hematological family history. These data are collected during the baseline visit and in case of disease progression or relapse.

### Medical and therapeutic data

At baseline and at time of disease progression, collected medical data include: ECOG performans status; the presence of end-organ damage (anemia, hypercalcemia, renal insufficiency or bone lesions; the presence of extramedullary disease; imaging; prognostic factors including albuminemia, serum beta2microglobulin, cytogenetic abnormalities including t(4;14), t(14,16), t(11;14), 17p deletion, 1q gain and 1p32 deletion); flow cytometry; percentage of bone marrow plasma cells; serum and urine amount of monoclonal protein; serum free light chain. The revised international scoring system will be determined at baseline and at time of disease progression [2]. At baseline, follow up visit and at time of disease progression, type of therapy, response to therapy and the duration of response are recorded.

#### Quality of life

At baseline, follow up visit and at time of disease progression, quality of life will be assessed by two standardized questionnaire, the medical outcomes study short form (SF-36) and the EuroQOL five dimensions survey (EQ-5D). The SF-36 survey is a general physical and mental health assessment used for comparison with general population norms [12]. It measures 36 items organized into 8 subscales and two summary scores, the physical component score and the mental component score. The EQ-5D facilitates pharmacoeconomic evaluation. It is a standardized survey measuring health-related quality of life and applicable to a wide range of health conditions and treatment [13].

#### Biological samples and functional assays

When available, tumor cells obtained from blood or bone marrow samples are collected at baseline and at time of disease progression. A part of the whole samples is used for functional studies and cryopreservation. Purified cells are stored as dry pellets for further DNA, RNA or protein analysis. Depending on the quantity of cells available, several analyses are planned. RNA analysis includes systematic quantification using digital gene expression sequencing (DGE Seq) of antiapoptotic genes implied in chemoresistance including Bcl-2, Bcl-XL or Mcl-1. As previously demonstrated, these antiapoptotic genes expression also predict sensitivity to targeted thrapy in MM [14]. Integrity of the p53 pathway, a strong prognostic factor influencing MM survival, is also assessed by DNA sequencing, FISH and by functional assay [15]. Single cell RNA sequencing analysis is perfomed using drop-seq technology and the commercialization of key-in-hand solution Chromium (10x Genomics Inc) [16]. Ex vivo functional assays are also perfomed on tumor cells and cells from microenvironment (i.e. drug testing, dependence to antiapoptotics proteins) [17, 18]. Sensitivity to targeted therapy is analyzed in regards to gene/protein expression and functional assays in order to develop predictive biomarkers. The BH3 profiling technique, predicting sensitivity to BH3 mimetics in MM, is also performed as previously described. Samples are stored in a unique biobank hosted by the CRCINA, INSERM, CNRS, Nantes, France.

### Data management

For each patient visit, data are collected on paper forms and entered into a central web-based database by the project workers and the medical team as previously described [19]. The system provides security with a protected access and complies with French safety policy. The data management team performs a data quality control every year. The local medical team is notified in case of discrepancy or incomplete data.

### Sample size and statistical analysis

In this study, the number of patients consulting for de novo or relapsed MM per year in the two centers determines the cohort size. Based on the study physicians’ experience, 10% of patients refuse to participate or don’t meet the inclusion criteria. According to the clinical activity of both centers, a sample size of sixty new inclusions per year is expected, meaning nearly 600 patients for a study period of 10 years. Interim analyses will be performed at 3 and 5 years. Confidence intervals, means, standard deviations and frequency distributions are calculated for all measures. For the epidemiological analyses, this cohort will be compared to national/regional averages. Response to treatment is determined according to international criteria [20]. Responses will be compared using fisher exact testing regarding categories of interest. As a cohort, response rates will be reported *per protocol*. Progression-free-survival (PFS), defined as the time from treatment assignment to disease progression or death from any cause, is calculated using the Kaplan-Meier method from the first day of therapy. Overall survival (OS) is calculated using the Kaplan-Meier method from the date of diagnosis to date of death. Predictive models (i.e. random forest) are used for OS analysis. Corrections for multiple comparisons are used to control the occurrence of type I statistical errors. When performed, multiple comparisons will be corrected according to a Benjamini-Hochberg procedure. QoL analyses data will be analyzed on dimensions’ improvements further summarized by a Pareto Classification of Health Change [21] The EQ-VAS and utility scores will be analyzed using a mixed linear modelling in case of sufficient data. A Factorial Analysis of Mixed Data (FAMD) will be performed in order to illustrate QoL changes along with time, according to treatment strategies and/or responses. Missing data will be reported in results; patients with recurrent lacking data will be investigated and may be discarded from further analyses. For multivariate analyses, in case of several missing data and not to impede power of analyses, an imputation will be performed as follow (and explicitly explained in the coming papers): for every variable, only those with no more than 10% of missing data will be imputed, in order not to introduce bias in imputation. The multiple imputations will be performed according Expected-Maximization alogorithm with Bootstrap, as implemented in the Amelia II package [22]. All analyses are performed with R. Kaplan-Meier survival curves are used to compare follow-up data. Significance levels will be standardly of 5%, otherwise specified. According to a cohort structure, all statistical comparisons will be 2-sided, with a bootstrapped 95% confidence interval of results. Logistic regression or Cox regression adjusts for patient characteristics and is used for survival analysis for different groups of patients. Depending on the statistical power available, predictive and interaction statistical models are used (i.e random forest, classification trees,

## Discussion

The MYRACLE study systematically and prospectively collect integrative data (socio-economic, clinical, prognostic, imaging, treatment, quality of life) from patients diagnosed with multiple myeloma. For each patient, these data are collected before and after subsequent anti myeloma therapies. At these different time points, tumor cells from patients are collected and stored in order to perform analysis dedicated to better understand drug resistance and clonal evolution. This long-term cohort gives us a unique opportunity to integrate this large variety of data in the context of real –life myeloma care. The recruitment is expected to start by January 2019. The MYRACLE study is part of the ILIAD (Imaging and Longitudinal Investigations to Ameliorate Decision-making in MM and breast cancer) project, supported by the French institute of cancer INCA (Institut National du Cancer). This integrative cohort will give us the ability to determine biomarkers influencing response to therapy, to evaluate the impact of each therapy on clonal evolution and on major resistance factors such as apoptosis dysregulation and p53 network. The analysis of response following each line of therapy, cost and quality of life will also provide important pharmacoeconomic data. The MYRACLE cohort is also designed to facilitate collaboration with investigators, institutions, cooperative groups and pharmaceutical companies working in the MM area.

## Data Availability

There are no data available as this is a study protocol.

## Abbreviations

CAR: Chimeric antigen receptors
CNRS: Centre National pour la Recherche Scientifque
CRCINA: Centre de Recherche en Cancérologie Nantes Angers
ECOG: Eastern cooperative oncology group
INSERM: Institut national de la Santé et recherché Médicale
MM: Multiple myeloma
MYRACLE: Myeloma resistance and clonal evolution
QoL: Quality of life
